# A New Therapeutic Agent

**Published:** 1888-08

**Authors:** 


A NEW THERAPEUTIC AGENT.—Our medical friend, of Panola, Ga., Dr. Lallerstidt, kindly sent us a sample of a plant the name of which he does not know, which he has found to be a useful remedy in diarrhœa and dysentery. We have not found time to investigate its botanical name and character. Dr. L. has, however, sent a sample to Parke, Davis & Co., with a request to name and classify the plant, and will report the same to our journal. It is well for our medical brethren in the country to inquire into the properties of plants that acquire a reputation for useful properties as medicines among the common people. Many of our most valuable indigenous remedies have thus come up from the masses.

DR. L. L. CLEMENT writes: A great many people up this way are suffering with a painful disorder of the stomach (or in the region of the stomach) which don't partake of the character of any especia disease of which I am acquainted. The pain radiates across the hypochondriac and epigastric regions very much like bilious colic or gastralgia; the tongue has a brownish coat and is very red where it has cleared off, which, apparently, would indicate irritability of the stomach ; but there is no nausea or vomiting ; appetite generally good ; the temperature, in some cases, reaches 103¾ degrees in the evenining ; there is not as much constitu'ional dis'urbance as the fever would indicate ; in the region of the stomach is sore on pressure ; there is an inability to get a long breath or cough as it causes a catch like a pluretic stitch. In a case or two there was an acute neuralgic pain in the neck or shoulder be-before the pain or soreness was located in the stomach. Patients are generally confined to bed or house about a week ; fever lasts about a week or two weeks; soreness in stomach two or three weeks. I call the trouble "gastric fever." Please write me what you think it is, also treatment. I just have a curiosity to know, not that it is a dangerous disease, but a troublesome one. I treat the trouble symptomatically: A mercurial purge, an opiate for pain, anti-dyspeptics, etc.

[We regard the disorder above described by Dr. Clement as one of the phases of hepatic congestion not unfrequently met with at the approach of the hot season in malarious regions. Answering to certain hepatic disorders described by Eberle as pertaining to the early spring and summer, especially in southern latitudes. In the hot and wet weather alternating with cool nights, we not unfrequently meet with what he terms pneumonia billiosa ; sometimes acute or subacute hepatitis; sometimes epidemic jaundice; all phases of the same general condition. The cases referred to by Dr. Clements present unmistakable symptoms of hepatic conjestion or engorgement involving the duodenum—the mucous coat of which is in a hyperæmic or congested state, obstructing the free flow of bile through the common bile duct, giving rise to tenderness or pain in the epigastric region, which is sensibly felt in breathing, in consequence of movements imparted by the diaphragm. The liver when thus engorged is enlarged and tender, and contributes to the embarrassment of the breathing The treatment indicated is the disgorgement of the liver and duodenum by broken doses of calomel and ipecac, rest in bed, revulsives to the epigastric and hypochondriac regions, with quinine at morning; and where fever exists, small and oft repeated doses of aconite in the afternoon, and very low diet. Opiates if the pain be severe.

